# Pattern of distant metastases in inflammatory breast cancer - A large-cohort retrospective study

**DOI:** 10.7150/jca.34572

**Published:** 2020-01-01

**Authors:** Zheng Wang, Mo Chen, Junjie Pan, Xuan Wang, Xiao-Song Chen, Kun-Wei Shen

**Affiliations:** 1 Comprehensive Breast Health Center, Ruijin Hospital, Shanghai Jiao Tong University School of Medicine, Shanghai 200025, China.; 2 Cancer Metastasis Institute, Fudan University, Shanghai 200040, China.

**Keywords:** Inflammatory breast cancer, metastasis, molecular subtype, prognosis, SEER

## Abstract

Inflammatory breast cancer (IBC) is a fatal form of breast cancer. IBC patients present with unfavorable prognosis mainly attributable to high risk of distant metastasis. Thus, in this cohort study, we aimed to explore metastatic profiles of different molecular subtypes of IBC and elucidate the clinical and prognostic characteristics among different metastatic sites. Patients diagnosed as IBC between 2010 and 2016 were identified from the Surveillance, Epidemiology and End Results (SEER) database. Chi-square tests were performed to compare metastatic distribution among different molecular subtypes. We further used odds ratio calculation to analyze the combined metastatic patterns. Kaplan-Meier methods and multivariate Cox regression models were applied to analyze survival data among different metastatic organs. In total, we enrolled 635 IBC patients between 2010 and 2014 as the training cohort and 242 IBC patients between 2015 and 2016 as the validation cohort, All the included patients were recorded with known metastatic status, follow-up data and molecular subtype. In the present study, we elaborated the following three points: (1) Elucidating the distribution of single-organ metastases in IBC. Bone and brain were the most and least common metastatic lesions for all subtypes of IBC, separately. (2) Clarifying the combined metastatic patterns and tendency of co-metastases. Bi-organ metastasis occurred most frequently among all combined metastases. Several combinations, such as liver and bone, lung and brain, were preferential for bi-organ metastasis. (3) Analyzing prognostic values of single-organ and bi-organ metastases. All single-organ distal metastases were independent risk factors indicating an unfavorable prognosis. In conclusion, our results would provide more information for clinical decision and future studies.

## Introduction

Breast cancer is the most frequent malignancy and the second most frequent cause of cancer related death in women 1. Inflammatory breast cancer (IBC), a locally advanced neoplasm, accounts for 1% to 2% of all invasive breast cancers 2. Despite its rarity, IBC contributes to a disproportionate number of breast cancer specific mortality 3. Due to tumor infiltration of the dermal lymphatics, the patients develop rapid onset of characteristic skin changes from the time of confirmed diagnosis 4. Thus, it is crucial to make an accurate diagnosis and start rapid approach to treatment for this fatal malignancy.

By far, management of IBC is based on multimodality therapy which includes surgery, chemotherapy and radiation therapy 5, 6. Under this circumstance, IBC patients have markedly poorer prognosis compared with non-inflammatory breast cancer, with under 55% of 5-year overall survival rate 7. This disappointing prognostic data mainly attributes to high risk of distant metastasis and locoregional recurrence 8. Surprisingly, 85% of IBC patients had regional lymph node invasion, and over 30% present with distant metastasis at the time of diagnosis 9. Bone is a common metastatic organ for IBC, as well as visceral sites including lung and liver 10. Previous studies suggested the prognostic difference between patients with bone metastasis and visceral metastasis 11, 12. Also, IBC patients have a high risk of developing brain metastasis, which usually portends a frustrating prognosis with a median survival less than six months 13.

However, the metastatic pattern of IBC still needs further investigation. And the prognostic ending of diverse metastatic lesions needs to be elaborated more clearly. Therefore, it would be vital to illustrate the metastatic distribution for better clinical decision. In the present cohort study, we aimed to explore metastatic profiles of IBC, by reviewing data from the SEER database. And we also intended to elucidate the clinical and prognostic characteristics among different metastatic sites.

## Methods

### Cohort population

A retrospective cohort research was performed by using data from the Surveillance, Epidemiology and End Results (SEER) national database. The SEER*Stat software (Version 8.4, National Cancer Institute, Washington DC, USA) was used to access the database and a data-use agreement was signed for this study.

**Figure 1:**
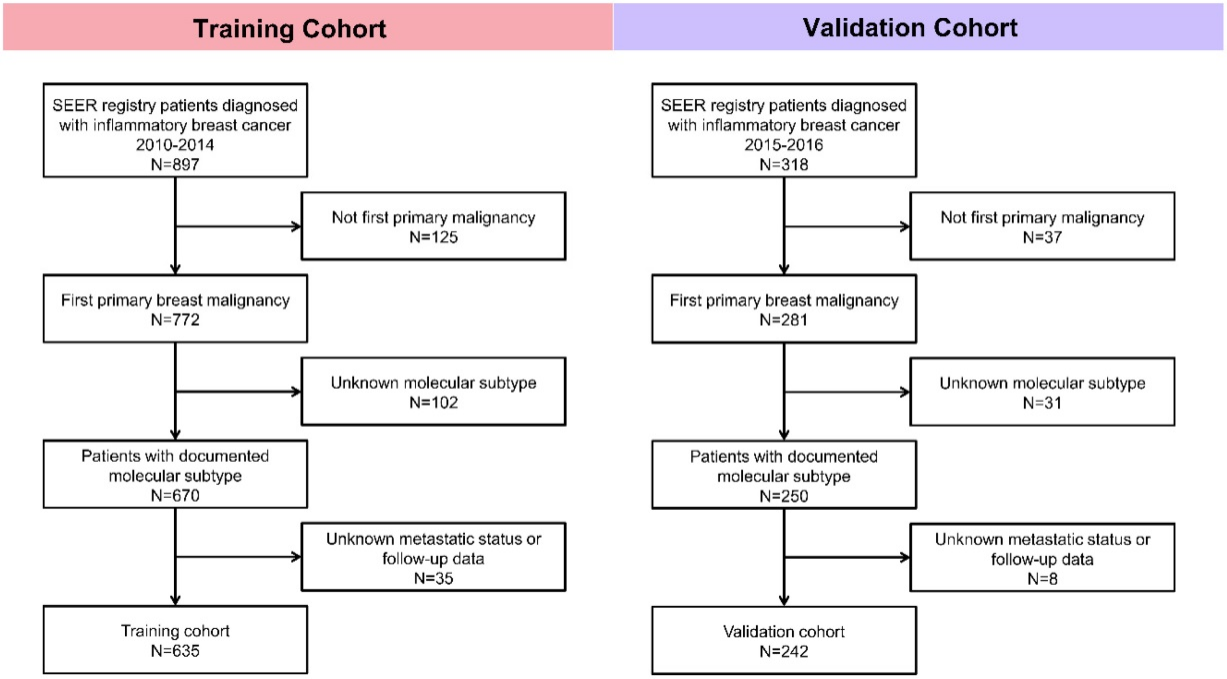
Flowchart of patient selection in this study.

Patients were enrolled in this study according to the following inclusion and exclusion criteria. Inclusion criteria: (1) Patients were diagnosed with inflammatory breast cancer; (2) Breast cancer was the first primary malignancy. Exclusion criteria: (1) Metastatic status was unknown; (2) Follow-up data was missing; (3) Information about molecular subtype was unknown. Patients diagnosed with IBC between 2010 and 2014 were studied as the training cohort, and IBC cases between 2015 and 2016 were assigned to the validation cohort. The detailed procedure of cohort selection was outlined (Fig. 1).

In this study, we intended to identify distant metastatic pattern of inflammatory breast cancer. And the SEER database contained metastatic information including bone, brain, liver, lung, and distant lymph nodes (DL), which could basically cover extensive metastatic sites of IBC.

### Statistical analysis

Patients' demographic and clinical characteristics were summarized by descriptive statistics. Chi-square tests were applied to compare metastatic distribution among different molecular subtypes. Frequency distribution among different metastatic sites was analyzed by odds ratio calculation. Overall survival (OS) and cancer-specific survival (CSS) among different metastatic organs were analyzed by Kaplan-Meier methods and log-rank tests. Independent prognostic risk factors were further assessed by multivariate Cox proportional hazards models. All tests were two-sided and P<0.05 indicated statistical significance. All statistical analyses were conducted by GraphPad Prism 6 (GraphPad Software, San Diego, CA, USA) and SPSS 22.0 (SPSS Inc. Chicago, IL, USA).

## Results

### Patient characteristics

In total, we selected 635 patients with the diagnosis of inflammatory breast cancer in the training cohort and 242 patients in the validation cohort. In the training cohort, 224 cases (35.3%) were HR-positive/HER2-negative, 133 cases (20.9%) were HR-positive/HER2-positive, 109 cases (17.2%) were HR-negative/HER2-positive, and 169 cases (26.6%) were triple negative breast cancer (TNBC). The baseline clinical characteristics were illustrated in Table 1. And in the validation cohort, 74 cases (30.6%) were HR-positive/HER2-negative, 52 cases (21.5%) were HR-positive/HER2-positive, 59 cases (24.4%) were HR-negative/HER2-positive, and 57 cases (23.6%) were triple negative breast cancer (TNBC) (Supplementary Table 1).

Demographic and clinicopathological parameters including marital status, race, tumor size, and regional lymph node invasion showed significant differences between the metastatic group and non-metastatic group. Compared to non-metastatic group, metastatic group tended to have higher rate of unmarried status, lower incidence of white race, larger tumor size and higher frequency of regional lymph node invasion. As for therapies, metastatic patients received less surgery, chemotherapy or radiation therapy than non-metastatic patients.

Among 635 enrolled cases in the training cohort, 224 cases (35.3%) were reported with distant metastasis. According to the obtained information from SEER database, the five metastatic organs (bone, brain, liver, lung and DL) accounted for 93.8% (210/224) of all metastatic IBC patients. Bone and brain were the most and least common metastatic organs, accounting for 57.6% (129/224) and 6.3% (14/224), respectively. In the validation cohort, 80 cases (33.1%) had distant metastasis, and bone and brain were the most and least common metastatic organs, accounting for 60.0% (48/80) and 7.5% (6/80), respectively.

### Metastatic pattern

**Figure 2:**
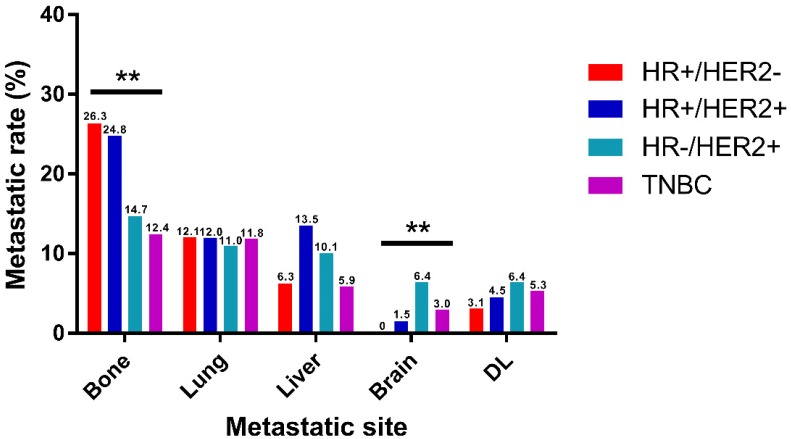
Distribution of distant metastatic organs according to molecular subtype. DL, distant lymph node. (*P<0.05, **P<0.01, ***P<0.001)

Based on the molecular subtype, IBC were classified into HR+/HER2-, HR+/HER2+, HR-/HER2+ and TNBC for metastatic distribution comparison. It is clearly shown in the two cohorts that bone was the most frequent metastatic organ and brain was the least frequent of all subtypes of IBC, separately (Fig. 2, Supplementary Fig. 1). Notably, there were some differences in patterns of metastasis among different subtypes in the training cohort. Incidence rate of bone metastasis was highest in HR+/HER2- (26.3%) and lowest in TNBC (12.4%). And the rate of brain metastasis was lowest in HR+/HER2- (0%) and highest in HR-/HER2+ (6.4%). And different subtypes of IBC showed no significantly diverse intends to lung, liver, and DL metastasis. The similar trends were observed in the validation cohort that incidence rate of bone metastasis was highest in HR+/HER2- (25.7%) and rate of brain metastasis was highest in HR-/HER2+ (6.8%).

### Combination of metastases

**Figure 3:**
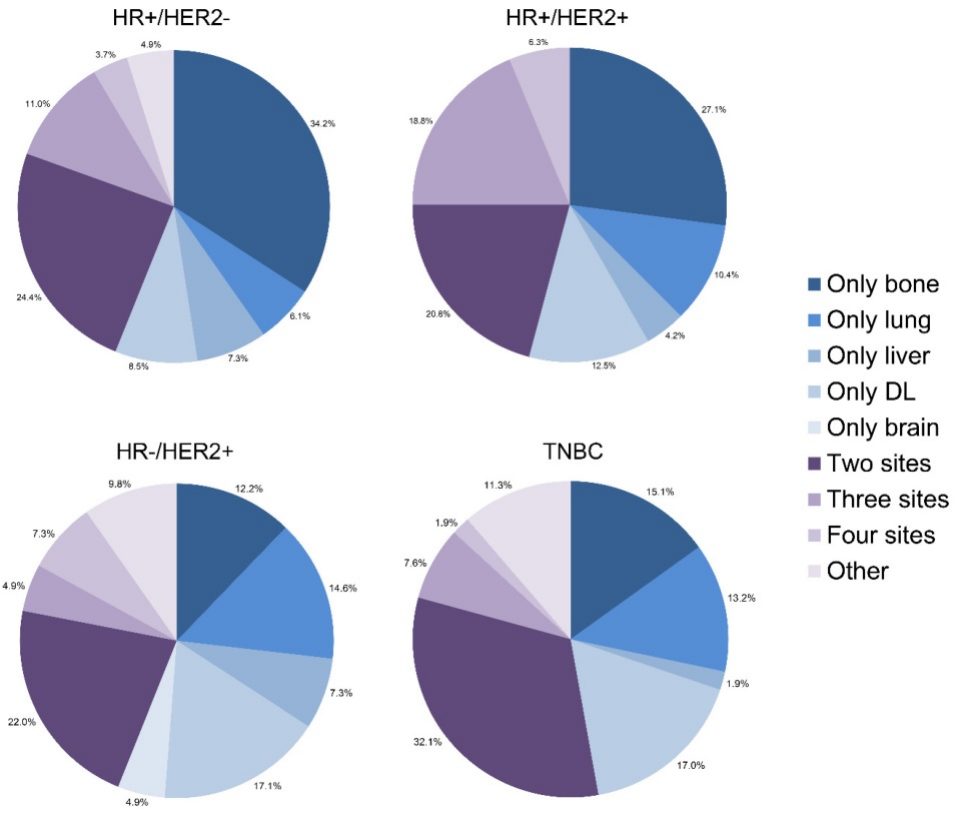
Relative rates of single and combined metastatic sites in different molecular.

Many patients developed more than one metastatic site simultaneously or sequentially. We drew pie charts to illustrate proportions of each single metastasis and combined metastatic patterns among all subtypes of IBC (Fig. 3, Supplementary Fig. 2). It is shown that bone and DL were two leading lesions as a single metastatic site for IBC. As for combination of metastases, bi-site pattern (HR+/HER2-: 24.4%, HR+/HER2+: 20.8%, HR-/HER2+: 22.0%, TNBC: 32.1%) was dominantly higher than tri-site (HR+/HER2-: 11.0%, HR+/HER2+: 18.8%, HR-/HER2+: 4.9%, TNBC: 7.6%) and tetra-site pattern (HR+/HER2-: 3.7%, HR+/HER2+: 6.3%, HR-/HER2+: 7.3%, TNBC: 1.9%) in the training cohort. The results were further confirmed in the validation cohort.

**Figure 4:**
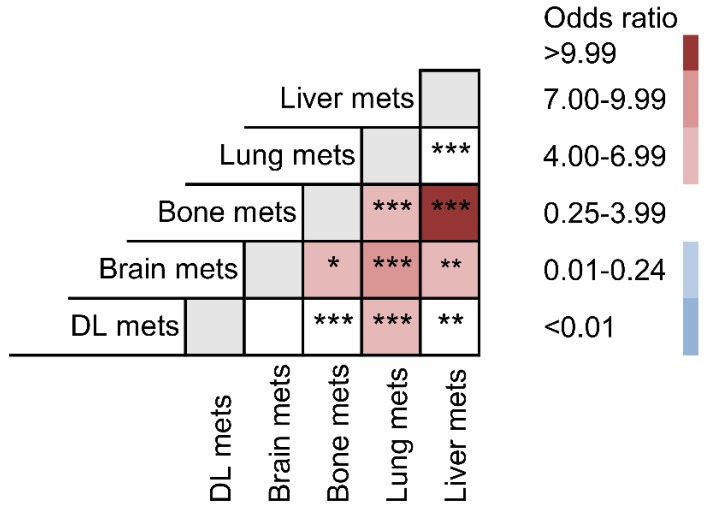
Odds ratio comparison among different metastatic combinations.

For a better understanding of the interaction among these metastatic lesions, odds ratio of each possible combination between all five organs was compared (Fig. 4). Liver metastasis preferentially intended to co-metastasize with bone metastasis (OR: 11.135) and brain metastasis (OR: 6.632). Their metastatic combinations were far more common than any other co-metastasis with liver. Lung metastasis was also specially related to brain (OR: 8.132), bone (OR: 6.920) and DL metastasis (OR: 4.597).

### Survival

**Figure 5:**
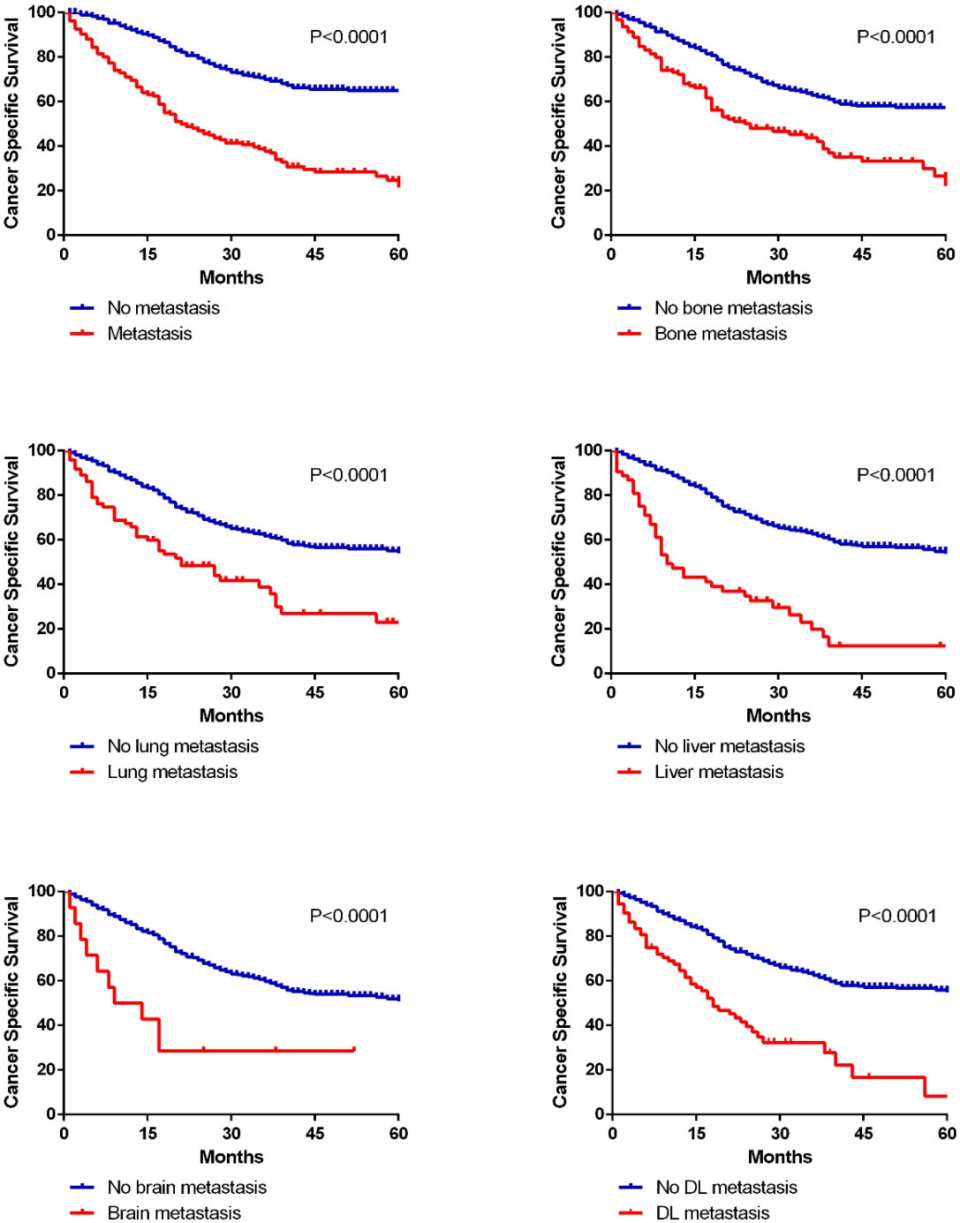
Kaplan-Meier curves of cancer specific survival in patients according to metastatic status.

In this cohort research, 290 deaths (45.7%) were observed among 635 patients. We calculated 1-year OS and CSS for patients with different metastatic lesions (Table 2). And there were extraordinarily large differences (P<0.001) in 1-year OS and CSS between patients with or without metastasis in all five organs (OS: bone 67.4% vs 84.6%, lung 60.0% vs 83.9%, liver 45.3% vs 84.4%, brain 50.0% vs 81.8%, DL 60.5% vs 83.9%; CSS: bone 71.3% vs 87.2%, lung 65.3% vs 86.4%, liver 49.1% vs 87.1%, brain 50.0% vs 84.7%, DL 63.2% vs 86.8%). Kaplan-Meier curves were applied to analyze the survival data of CSS more intuitively in both cohorts (Fig. 5, Supplementary Fig. 3).

Moreover, multivariate Cox regression models were performed to identify whether metastatic status was an independent prognostic factor (Table 3, Supplementary Table 2). After adjusting for molecular subtype, age, race, marital status, grade, tumor size, regional lymph node invasion and therapies, we found that different metastatic sites were all related to worse OS (bone: HR 1.989, 95%CI 1.487-2.660; lung: HR 1.929, 95%CI 1.395-2.667; liver HR 4.008, 95%CI 2.823-5.690; brain HR 2.707, 95%CI 1.341-5.465; DL HR 2.178, 95%CI 1.563-3.036), as well as worse CSS (bone: HR 2.081, 95%CI 1.527-2.838; lung: HR 1.970, 95%CI 1.395-2.780; liver HR 4.418, 95%CI 3.064-6.370; brain HR 3.213, 95%CI 1.583-6.519; DL HR 2.435, 95%CI 1.725-3.437). And in the validation cohort, all the different metastatic sites were identified as independent risk factors for unfavorable OS and CSS.

**Figure 6:**
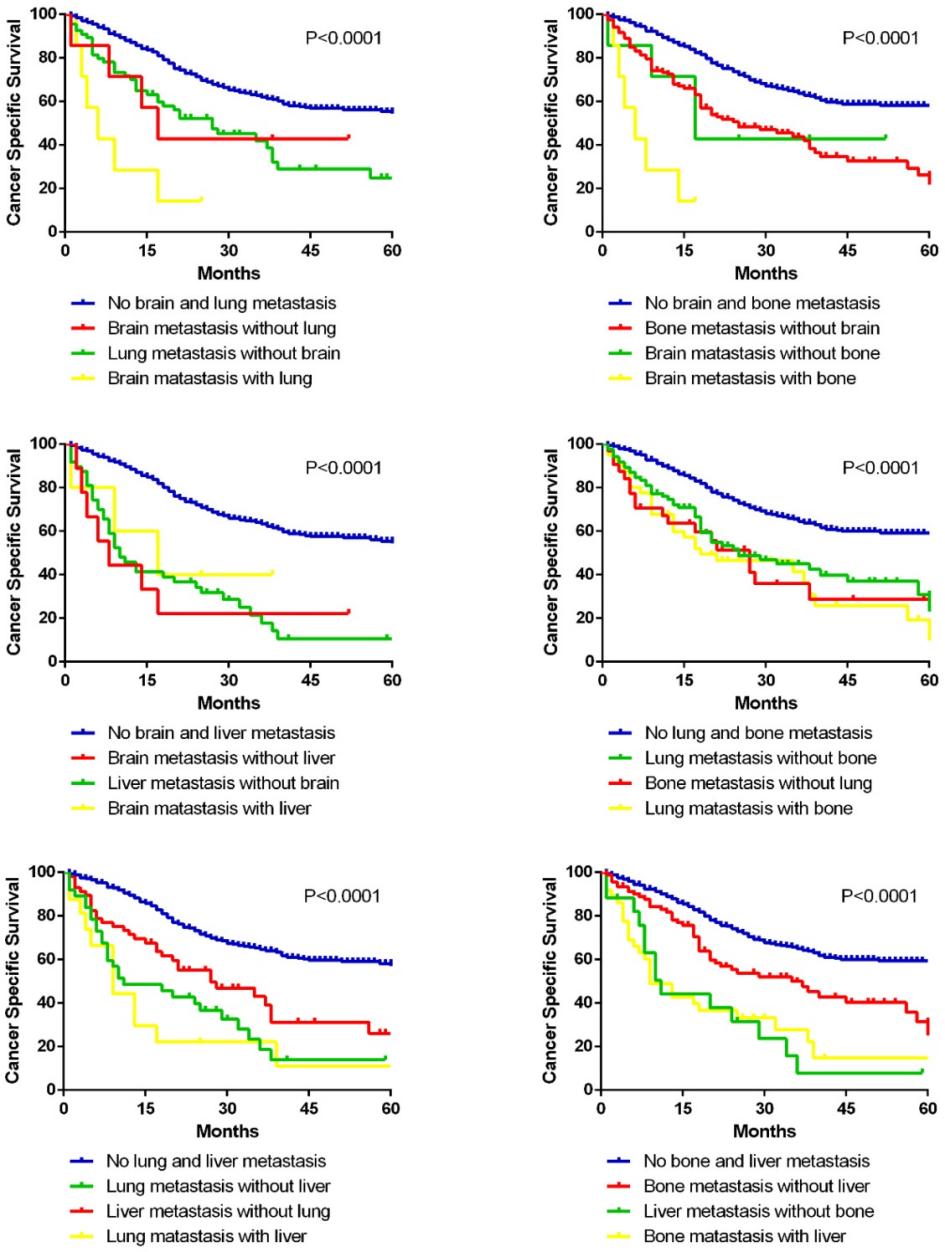
Kaplan-Meier curves of cancer specific survival in patients with different bi-site metastatic patterns.

In addition, prognostic differences between different bi-site metastatic patterns were compared among the four solid organs (bone, lung, brain and liver) by Kaplan-Meier methods (Fig. 6). It is clearly shown that brain metastasis combined with lung or bone, and lung metastasis combined with liver ended up worse prognosis than the separated single metastasis. Interestingly, as for the combined metastasis of brain and liver, also lung and bone, bi-site metastasis resulted in no worse ending than the separated single metastasis.

## Discussion

Inflammatory breast cancer shows unfavorable prognosis mainly attributable to early distant metastasis 14, 15. It is important to have a clear understanding of its metastatic pattern. In our research, the following three points were mainly elaborated: (1) Elucidating the distribution of single-organ metastases in IBC; (2) Clarifying the combined metastatic patterns and tendency of co-metastases; (3) Analyzing prognostic values of single-organ and bi-organ metastases. To our knowledge, this SEER-based research is the first comprehensive, large-cohort research focusing on the distal metastatic pattern of IBC and considering different molecular subtypes as separate entities. And we hope our results can be beneficial for treatment selection and clinical research.

As previous studies mentioned, the prevalence in breast cancer patients developing bone metastasis is extremely high. That is to say, bone is the most common metastatic lesion in both IBC and non-IBC patients 16, 17. Consistent with the conventional views, we found that bone and brain were the most and least common metastatic organs for all subtypes of IBC, separately. Several differences existed among different molecular subtypes in bone and brain, but not in lung, liver or DL. Incidence rate of bone metastasis was highest in HR+/HER2-, followed by HR+/HER2+, HR-/HER2+ and lowest in TNBC.

And we also found that the rate of brain metastasis was highest in HR-/HER2+, followed by TNBC, HR-/HER2+ and lowest in HR+/HER2-. Previous articles showed that TNBC had a significantly lower rate of bone metastasis than HR+/HER2- tumors 18, and that HR-/HER2+ patients had a higher risk of brain metastasis than HR+/HER2- patients 19, 20. These previous findings were supported by our research in IBC.

According to the baseline clinical characteristics, parameters including marital status, race, tumor size, and regional lymph node invasion showed significant differences between the metastatic group and non-metastatic group. Compared to non-metastatic group, metastatic group tended to have higher rate of unmarried status, lower incidence of white race, larger tumor size and higher frequency of regional lymph node invasion. As for therapies, metastatic patients received less surgery, chemotherapy or radiation therapy than non-metastatic patients. In consideration of these clinical parameters that could have influence on patient survival, we performed multivariate Cox regression models for further investigation. After adjusting for demographic, clinicopathological and therapeutic variables, we suggested that all single-organ metastases were independent risk factors for prognosis.

By far, previous studies have not focused on the combined metastatic patterns of IBC. Our findings showed that not any two metastatic organs were randomly combined for bi-site metastasis. For instance, liver metastasis had a preference for co-metastasizing with bone and brain. And lung was a common co-metastatic site for brain, bone and DL. Although the sequential information of co-metastatic organs was not provided by the SEER database, knowing the tendency of metastatic combinations would be beneficial for risk assessment and diagnostic screening in IBC patients with advanced stage. Additionally, we also analyzed the prognostic significance of different combinations with bi-organ metastases. However, not all bi-organ metastases had an unfavorable prognosis than the single-site metastases, which could be challenging for the conventional views. It could be partially explained that single-site metastasis may have already resulted in a fatal ending in some circumstances. And we hope further investigations could be conducted to better answer this phenomenon.

As far as we know, this cohort study is among the innovative work to summarize metastatic patterns of different molecular subtypes of IBC. As a retrospective research, several potential limitations need to be mentioned. First, since SEER database collected detailed information on distal metastatic site and molecular subtype from 2010, we only enrolled patients diagnosed with IBC between 2010 and 2016. Second, metastatic data extracted from the database is restricted to five organs (bone, lung, liver, brain and distant lymph node) and other distal sites are unclear. However, these five sites accounted for more than 90% of metastatic lesions and only a relatively small proportion of other sites may be ignored. Third, the metastatic information in this study was synchronous metastasis at the time of diagnosis, meaning that metachronous metastasis was not included. Briefly, on the basis of single-organ and bi-organ metastatic patterns, we recommend that the primary neoplasm and the first metastatic lesion could be taken into consideration for predictive prognostic model and optimal treatment choice.

In conclusion, in this SEER-based cohort study, we illustrated the metastatic patterns in different subtypes of IBC. Bone and brain were the most and least common metastatic organs for all subtypes of IBC, separately. Bi-organ metastasis occurred most frequently among all combined metastases. Several combinations, such as liver and bone, lung and brain, were preferential for bi-organ metastasis. And all single organ distal metastases were independent risk factors for prognostic prediction (adjusting for molecular subtype, therapy and other clinicopathological variables). Therefore, our results would provide more information for future study design and clinical decision.

## Supplementary Material

Supplementary figures and tables.

## Figures and Tables

**Table 1:** Baseline clinical characteristics of inflammatory breast cancer patients.

Characteristics	No metastasis		Metastasis		*P* value
	Number	%	Number	%	
**Subtype**					0.658
HR+/HER2-	142	34.5	82	36.6	
HR+/HER2+	85	20.7	48	21.4	
HR-/HER2+	68	16.5	41	18.3	
TNBC	116	28.2	53	23.7	
**Age**					0.158
<50	135	32.8	58	25.9	
51-65	204	49.6	127	56.7	
≥65	72	17.5	39	17.4	
**Marital status**					0.018
Married	206	50.1	87	38.8	
Unmarried	189	46.0	123	54.9	
Unknown	16	3.9	14	6.3	
**Race**					0.007
White	336	81.8	160	71.4	
Black	46	11.2	44	19.6	
Others*	29	7.1	20	8.9	
**Grade**					0.308
I	7	1.7	4	1.8	
II	94	22.9	44	19.6	
III	211	51.3	105	46.9	
IV	6	1.5	6	2.7	
Unknown	93	22.6	65	29.0	
**Size (cm)**					0.027
<2.0	39	9.5	20	8.9	
2.0-4.9	94	22.9	36	16.1	
≥5.0	135	32.8	64	28.6	
Unknown	143	34.8	104	46.4	
**Regional lymph node invasion**					0.019
N0	68	16.5	23	10.3	
N1	186	45.3	97	43.3	
N2	72	17.5	34	15.2	
N3	76	18.5	60	26.8	
NX	9	2.2	10	4.5	
**Surgery**					<0.001
Yes	320	77.9	77	34.4	
No	91	22.1	147	65.6	
**Chemotherapy**					<0.001
Yes	371	90.3	178	79.5	
No	40	9.7	46	20.5	
**Radiation therapy**					<0.001
Yes	213	51.8	54	24.1	
No	198	48.2	170	75.9	

*Others include American Indian, AK Native, Asian, and Pacific Islander. HR: Hormone Receptor; TNBC: Triple-Negative Breast Cancer.

**Table 2:** Survival analysis in diverse metastatic organs.

Parameter	1-year OS (%)	Univariate analysis		1-year CSS (%)	Univariate analysis	
		Log rank χ^2^ test	*P*		Log rank χ^2^ test	*P*
**Bone**						
No metastasis	84.6	22.612	<0.001	87.2	22.068	<0.001
Metastasis	67.4			71.3		
**Lung**						
No metastasis	83.9	30.528	<0.001	86.4	27.412	<0.001
Metastasis	60.0			65.3		
**Liver**						
No metastasis	84.4	61.944	<0.001	87.1	66.411	<0.001
Metastasis	45.3			49.1		
**Brain**						
No metastasis	81.8	12.199	<0.001	84.7	15.972	<0.001
Metastasis	50.0			50.0		
**Distant lymph nodes**						
No metastasis	83.9	30.786	<0.001	86.8	35.067	<0.001
Metastasis	60.5			63.2		


OS: Overall Survival; CSS: Cancer Specific Survival.

**Table 3:** Multivariate analyses of OS and CSS according to metastatic organs.

Variable	OS		CSS	
	HR (95% CI)	*P*	HR (95% CI)	*P*
No metastasis	Reference		Reference	
Bone metastasis	1.989 (1.487-2.660)	<0.001	2.081 (1.527-2.838)	<0.001
Lung metastasis	1.929 (1.395-2.667)	<0.001	1.970 (1.395-2.780)	<0.001
Liver metastasis	4.008 (2.823-5.690)	<0.001	4.418 (3.064-6.370)	<0.001
Brain metastasis	2.707 (1.341-5.465)	0.005	3.213 (1.583-6.519)	0.001
DL metastasis	2.178 (1.563-3.036)	<0.001	2.435 (1.725-3.437)	<0.001

Adjusted for molecular subtype, age, race, marital status, grade, tumor size, regional lymph node invasion and therapies. OS: Overall Survival; CSS: Cancer Specific Survival; HR: Hazard Ratio.

## References

[1] Siegel RL, Miller KD, Jemal A (2019). Cancer statistics, 2019. CA Cancer J Clin.

[2] Hance KW, Anderson WF, Devesa SS, Young HA, Levine PH (2005). Trends in inflammatory breast carcinoma incidence and survival: the surveillance, epidemiology, and end results program at the National Cancer Institute. J Natl Cancer Inst.

[3] Fouad TM, Barrera A, Reuben JM, Lucci A, Woodward WA, Stauder MC (2017). Inflammatory breast cancer: a proposed conceptual shift in the UICC-AJCC TNM staging system. Lancet Oncol.

[4] van Uden DJ, van Laarhoven HW, Westenberg AH, de Wilt JH, Blanken-Peeters CF (2015). Inflammatory breast cancer: an overview. Crit Rev Oncol Hematol.

[5] Saigal K, Hurley J, Takita C, Reis IM, Zhao W, Rodgers SE (2013). Risk factors for locoregional failure in patients with inflammatory breast cancer treated with trimodality therapy. Clin Breast Cancer.

[6] Abrous-Anane S, Savignoni A, Daveau C, Pierga JY, Gautier C, Reyal F (2011). Management of inflammatory breast cancer after neoadjuvant chemotherapy. Int J Radiat Oncol Biol Phys.

[7] Rueth NM, Lin HY, Bedrosian I, Shaitelman SF, Ueno NT, Shen Y (2014). Underuse of trimodality treatment affects survival for patients with inflammatory breast cancer: an analysis of treatment and survival trends from the National Cancer Database. J Clin Oncol.

[8] Cristofanilli M, Valero V, Buzdar AU, Kau SW, Broglio KR, Gonzalez-Angulo AM (2007). Inflammatory breast cancer (IBC) and patterns of recurrence: understanding the biology of a unique disease. Cancer-Am Cancer Soc.

[9] Walshe JM, Swain SM (2005). Clinical aspects of inflammatory breast cancer. Breast Dis.

[10] Kai M, Kogawa T, Liu DD, Fouad TM, Kai K, Niikura N (2015). Clinical characteristics and outcome of bone-only metastasis in inflammatory and noninflammatory breast cancers. Clin Breast Cancer.

[11] Niikura N, Liu J, Hayashi N, Palla SL, Tokuda Y, Hortobagyi GN (2011). Treatment outcome and prognostic factors for patients with bone-only metastases of breast cancer: a single-institution retrospective analysis. Oncologsit.

[12] Ahn SG, Lee HM, Cho SH, Lee SA, Hwang SH, Jeong J (2013). Prognostic factors for patients with bone-only metastasis in breast cancer. Yonsei Med J.

[13] Uemura MI, French JT, Hess KR, Liu D, Raghav K, Hortobagyi GN (2018). Development of CNS metastases and survival in patients with inflammatory breast cancer. Cancer-Am Cancer Soc.

[14] Wingo PA, Jamison PM, Young JL, Gargiullo P (2004). Population-based statistics for women diagnosed with inflammatory breast cancer (United States). Cancer Causes Control.

[15] Mamouch F, Berrada N, Aoullay Z, El KB, Errihani H (2018). Inflammatory Breast Cancer: A Literature Review. World J Oncol.

[16] Coleman RE, Rubens RD (1987). The clinical course of bone metastases from breast cancer. Br J Cancer.

[17] Coleman RE (2006). Clinical features of metastatic bone disease and risk of skeletal morbidity. Clin Cancer Res.

[18] Xiao W, Zheng S, Yang A, Zhang X, Zou Y, Tang H (2018). Breast cancer subtypes and the risk of distant metastasis at initial diagnosis: a population-based study. Cancer Manag Res.

[19] Brufsky AM, Mayer M, Rugo HS, Kaufman PA, Tan-Chiu E, Tripathy D (2011). Central nervous system metastases in patients with HER2-positive metastatic breast cancer: incidence, treatment, and survival in patients from registHER. Clin Cancer Res.

[20] Niwinska A, Murawska M, Pogoda K (2010). Breast cancer brain metastases: differences in survival depending on biological subtype, RPA RTOG prognostic class and systemic treatment after whole-brain radiotherapy (WBRT). Ann Oncol.

