# Supplementary material for: Pattern of distant metastases in inflammatory breast cancer - A large-cohort retrospective study
Source: J Cancer. 2020 Jan 1;11(2):292–300. doi: 10.7150/jca.34572 (PMC6930435; doi:10.7150/jca.34572)

Supplementary Table 1. Baseline clinical characteristics of inflammatory breast cancer patients in the validation cohort.

**Characteristics**	**No metastasis**		**Metastasis**		***P* value**
	**Number**	**%**	**Number**	**%**	
Subtype					0.521
HR+/HER2-	45	27.8	29	36.3	
HR+/HER2+	35	21.6	17	21.3	
HR-/HER2+	43	26.5	16	20.0	
TNBC	39	24.1	18	22.5	
Age					0.982
<50	42	25.9	21	26.3	
51-65	87	53.7	42	52.5	
≥65	33	20.4	17	21.3	
Marital status					0.917
Married	80	49.4	40	50.0	
Unmarried	74	45.7	37	46.3	
Unknown	8	4.9	3	3.8	
Race					0.447
White	118	72.8	55	68.8	
Black	28	17.3	19	23.8	
OthersΔ	16	9.9	6	7.5	
Grade					0.067
I	5	3.1	0	0	
II	31	19.1	21	26.3	
III	95	58.6	35	43.8	
IV	1	0.6	1	1.3	
Unknown	30	18.5	23	28.7	
Size (cm)					0.013
<2.0	13	8.0	4	5.0	
2.0-4.9	34	21.0	11	13.8	
≥5.0	65	40.1	23	28.7	
Unknown	50	30.9	42	52.5	
Regional lymph node invasion					0.072
N0	19	11.7	10	12.5	
N1	69	42.6	30	37.5	
N2	37	22.8	15	18.8	
N3	34	21.0	17	21.3	
NX	3	1.9	8	10.0	
Surgery					<0.001
Yes	126	77.8	19	23.8	
No	36	22.2	61	76.3	
Chemotherapy					0.029
Yes	146	90.1	64	80.0	
No	16	9.9	16	20.0	

Radiation therapy					<0.001
Yes	88	54.3	12	15.0	
No	74	45.7	68	85.0	

ΔOthers include American Indian, AK Native, Asian, and Pacific Islander. HR: Hormone Receptor; TNBC: Triple-Negative Breast Cancer.

Supplementary Table 2. Multivariate analyses of OS and CSS according to metastatic organs in the validation cohort.

**Variable**	**OS**		**CSS**	
	**HR (95% CI)**	***P***	**HR (95% CI)**	***P***
No metastasis	Reference		Reference	
Bone metastasis	3.106 (1.558-6.211)	0.001	3.521 (1.733-7.143)	0.001
Lung metastasis	1.114 (1.395-2.577)	0.014	1.789 (1.172-2.732)	0.007
Liver metastasis	1.835 (1.245-2.703)	0.002	1.789 (1.189-2.695)	0.005
Brain metastasis	1.427 (1.051-1.938)	0.023	1.368 (1.026-1.824)	0.033
DL metastasis	2.486 (1.163-5.317)	0.019	2.506 (1.128-5.567)	0.024

Adjusted for molecular subtype, age, race, marital status, grade, tumor size, regional lymph node invasion and therapies. OS: Overall Survival; CSS: Cancer Specific Survival; HR: Hazard Ratio.

Supplementary Fig 1. Distribution of distant metastatic organs according to molecular subtype in the validation cohort. DL, distant lymph node.

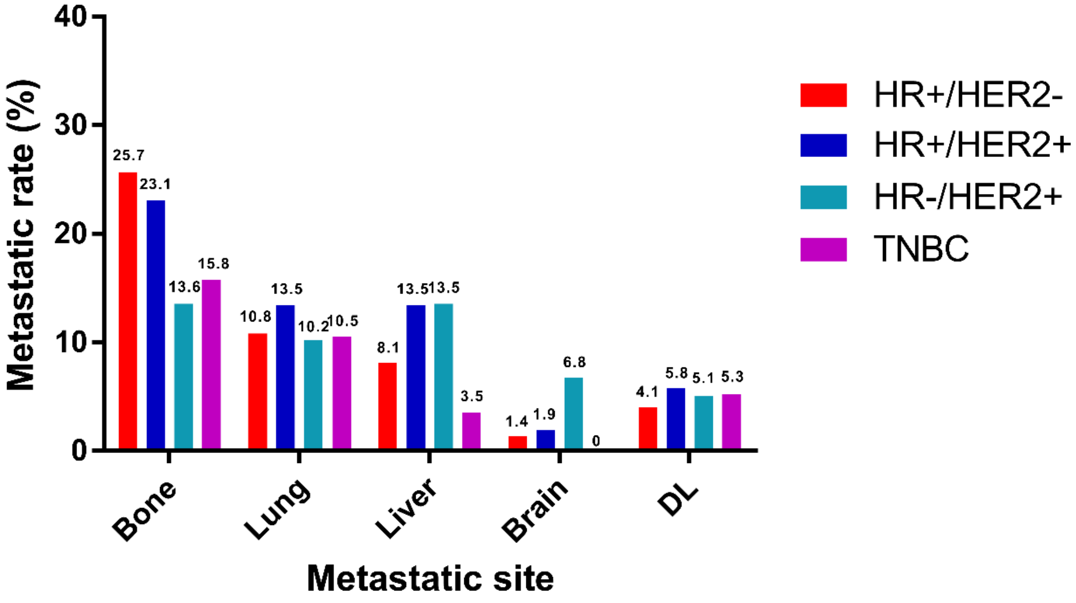

Supplementary Fig 2. Relative rates of single and combined metastatic sites in different molecular subtypes of inflammatory breast cancer in the validation cohort.

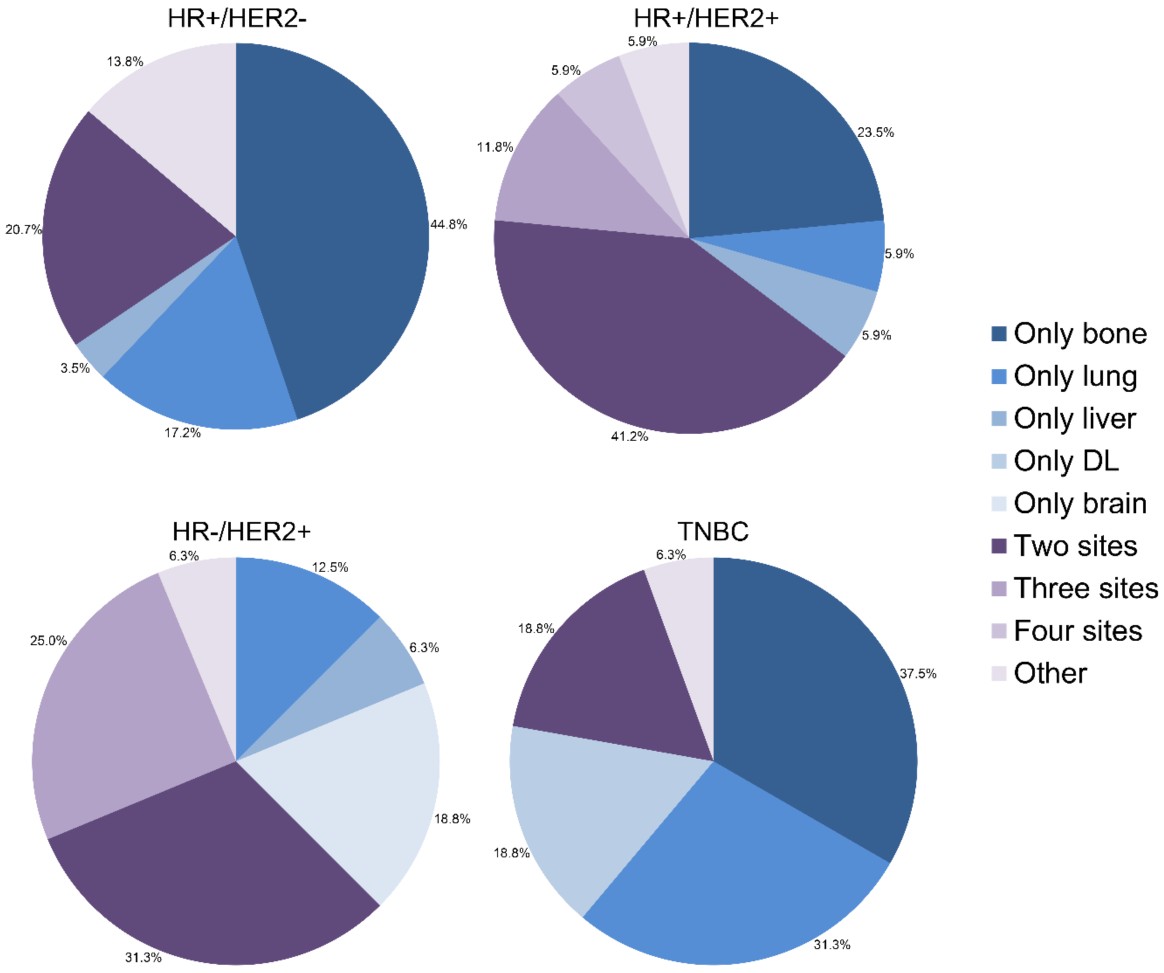

Supplementary Fig 3. Kaplan-Meier curves of cancer specific survival in patients according to metastatic status in the validation cohort.

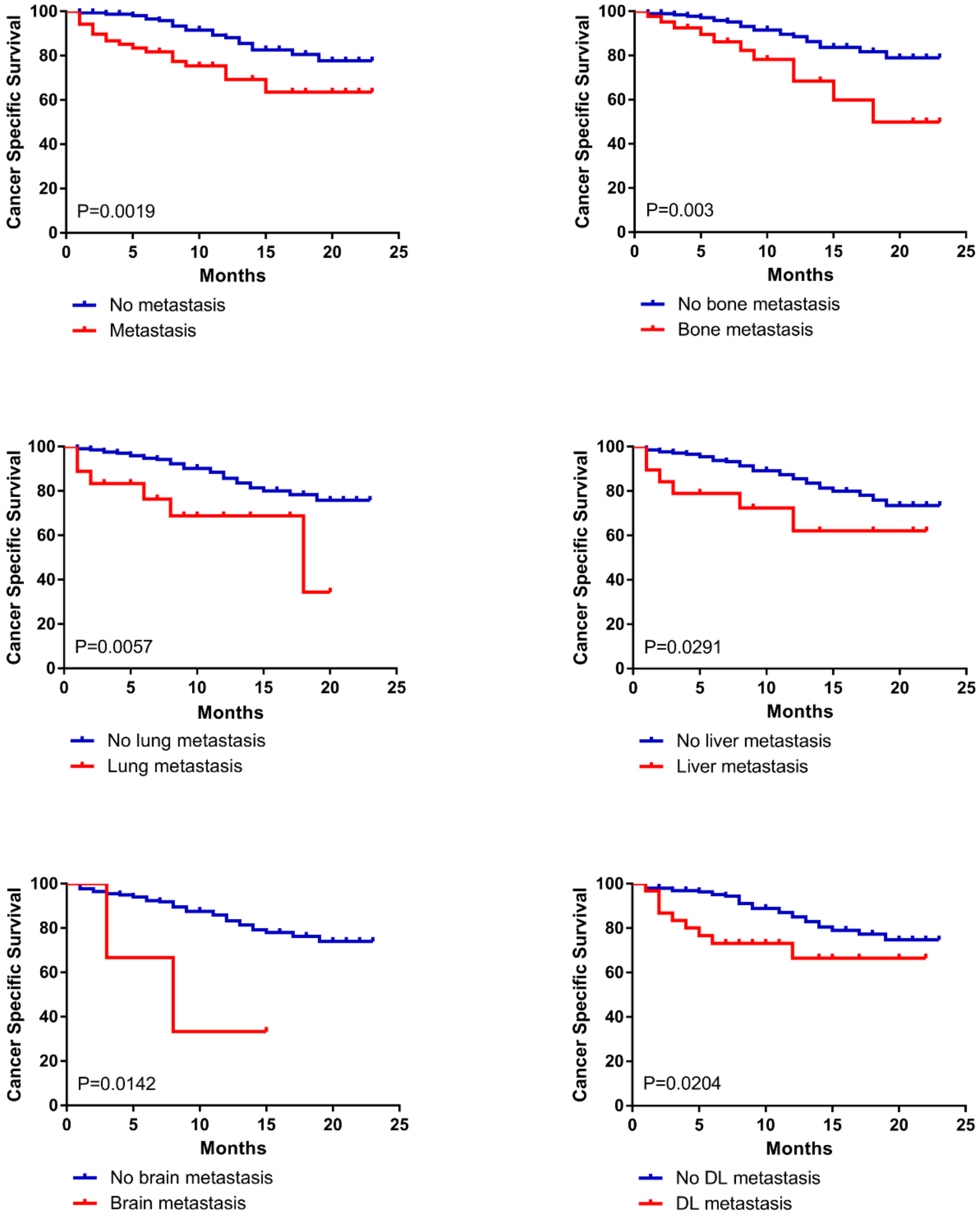

Supplement: Supplementary file 1 — Supplementary figures and tables. [file jcav11p0292s1.pdf]
